# Supplementary figures and images for: Synthesis of Daidzein Glycosides, α-Tocopherol Glycosides, Hesperetin Glycosides by Bioconversion and Their Potential for Anti-Allergic Functional-Foods and Cosmetics
Source: Molecules. 2019 Aug 16;24(16):2975. doi: 10.3390/molecules24162975 (PMC6721765; doi:10.3390/molecules24162975)

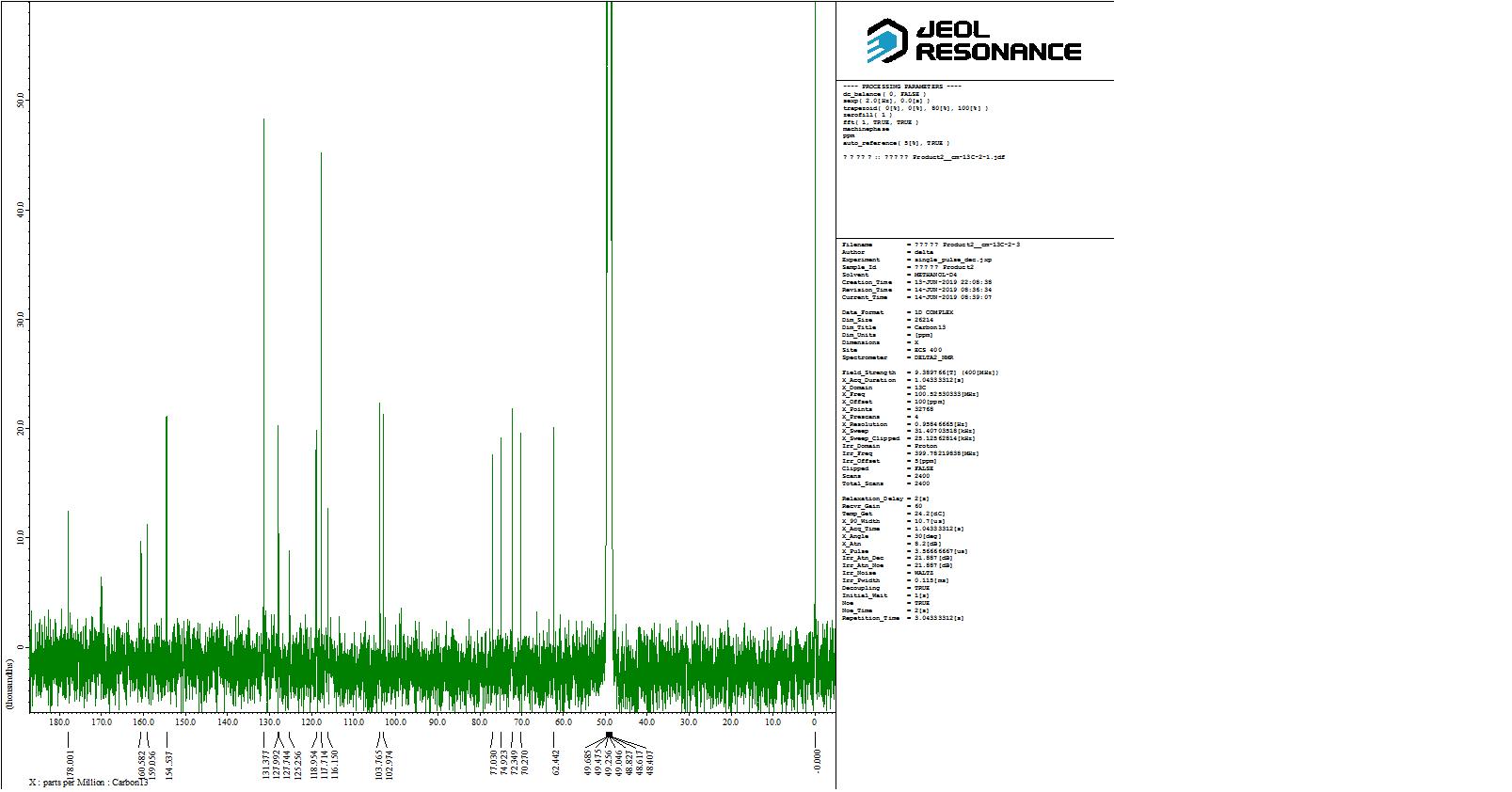

Supplement: Supplementary file 1 [file molecules-24-02975-s001.zip › supl/daidzein 4'-galactoside 13C NMR.jpg]

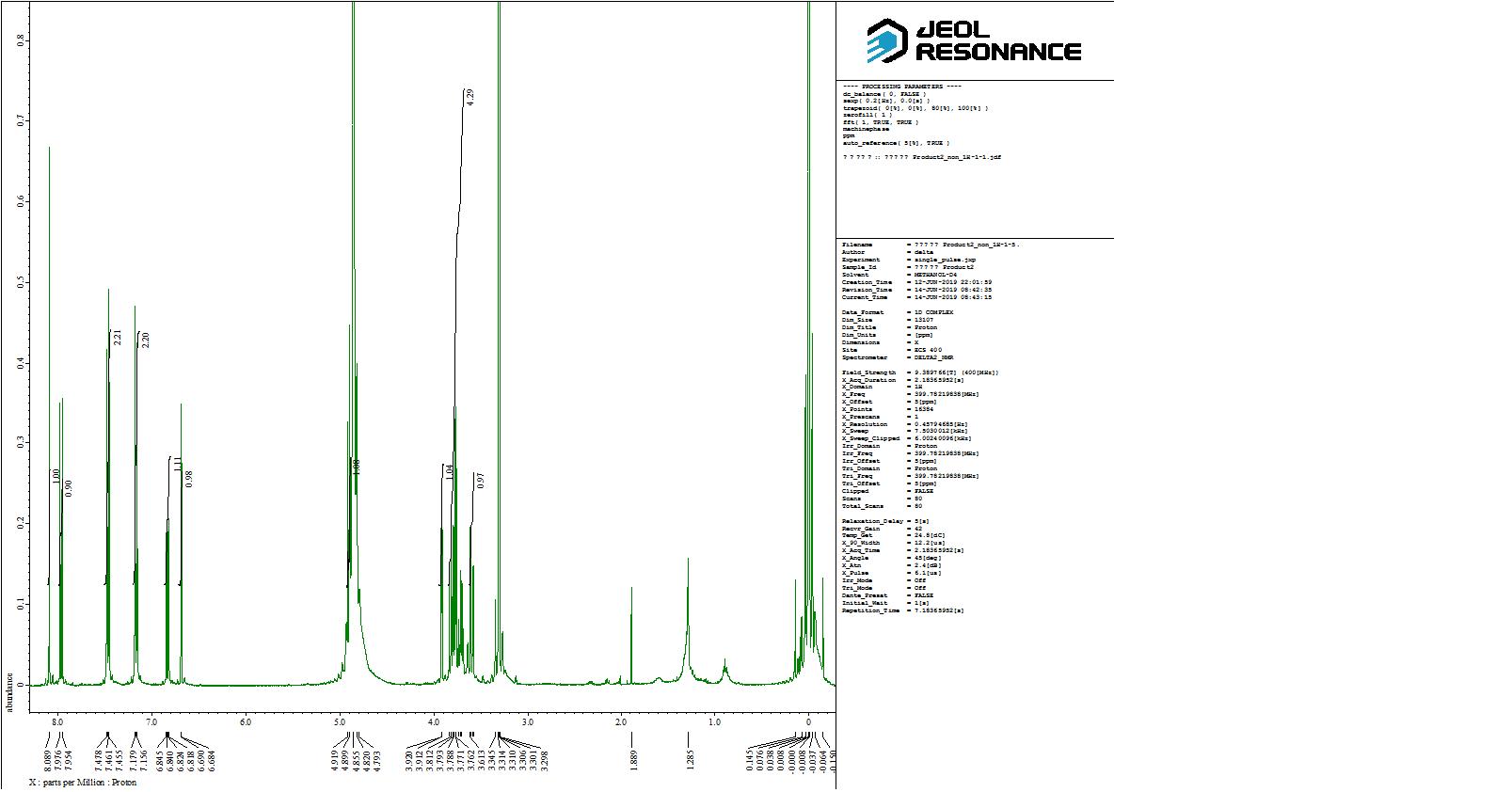

Supplement: Supplementary file 1 [file molecules-24-02975-s001.zip › supl/daidzein 4'-galactoside 1H NMR.jpg]

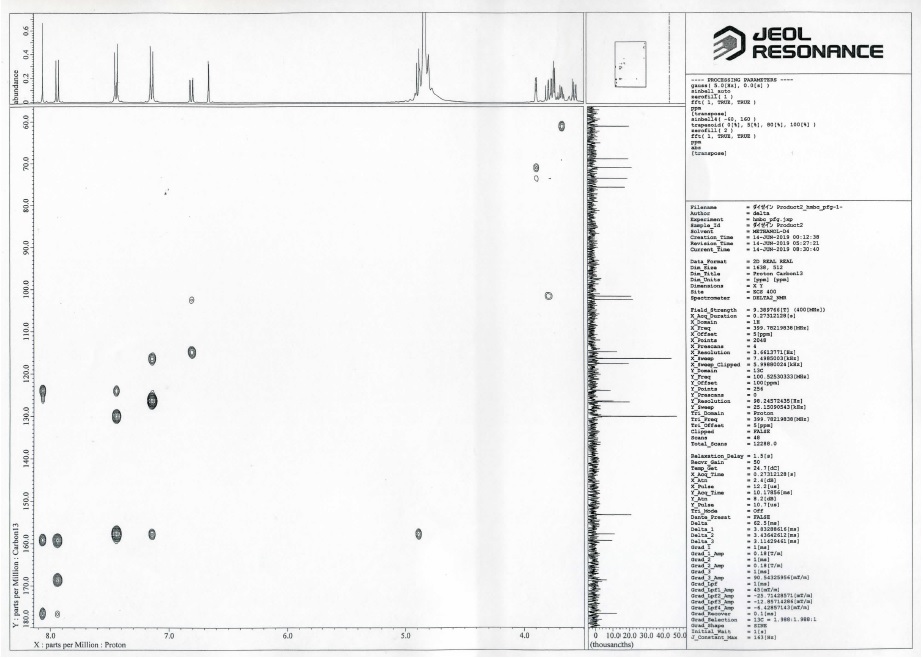

Supplement: Supplementary file 1 [file molecules-24-02975-s001.zip › supl/daidzein 4'-galactoside hmbc.jpg]

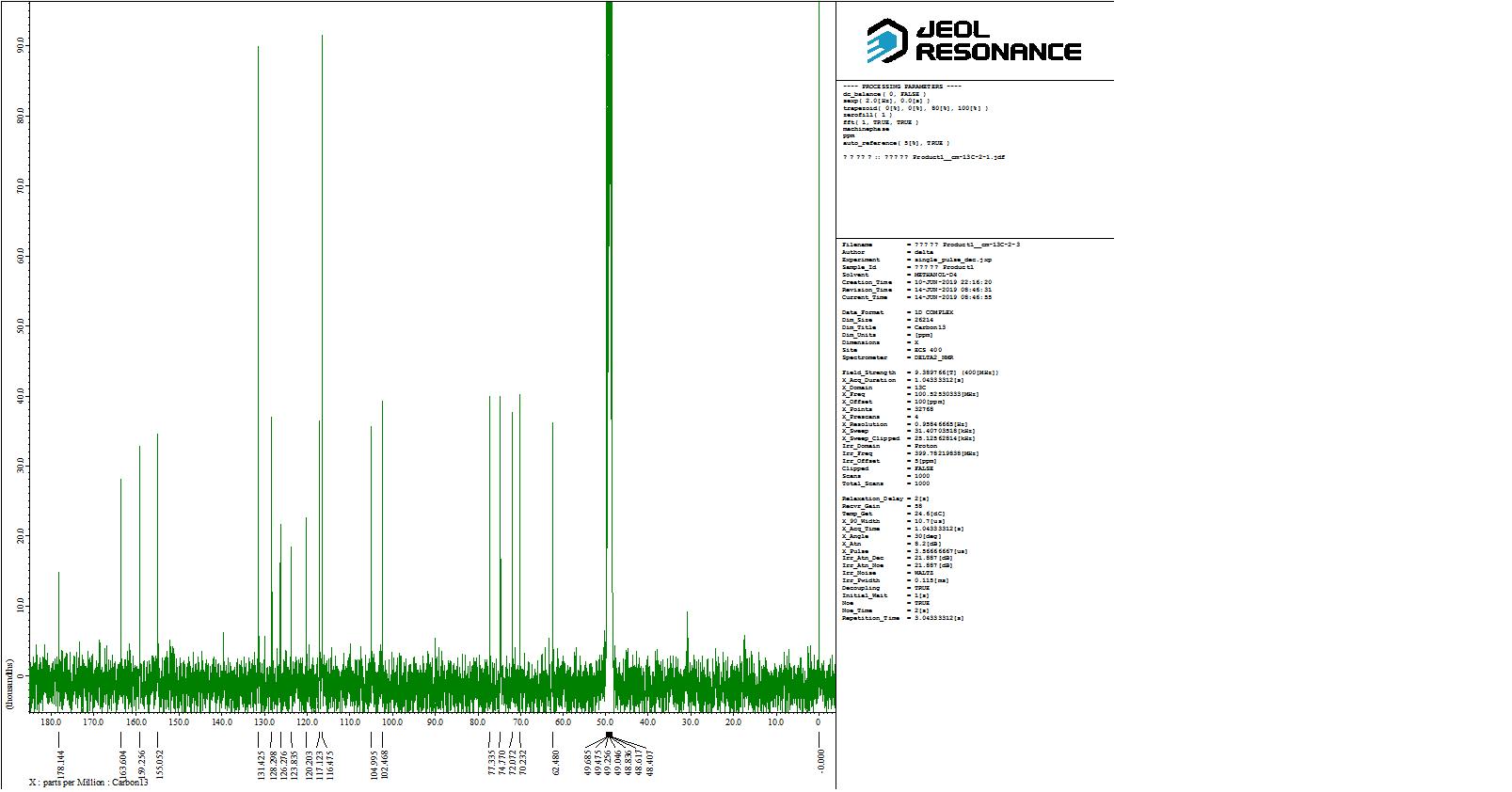

Supplement: Supplementary file 1 [file molecules-24-02975-s001.zip › supl/daidzein 7-galactoside 13C NMR.jpg]

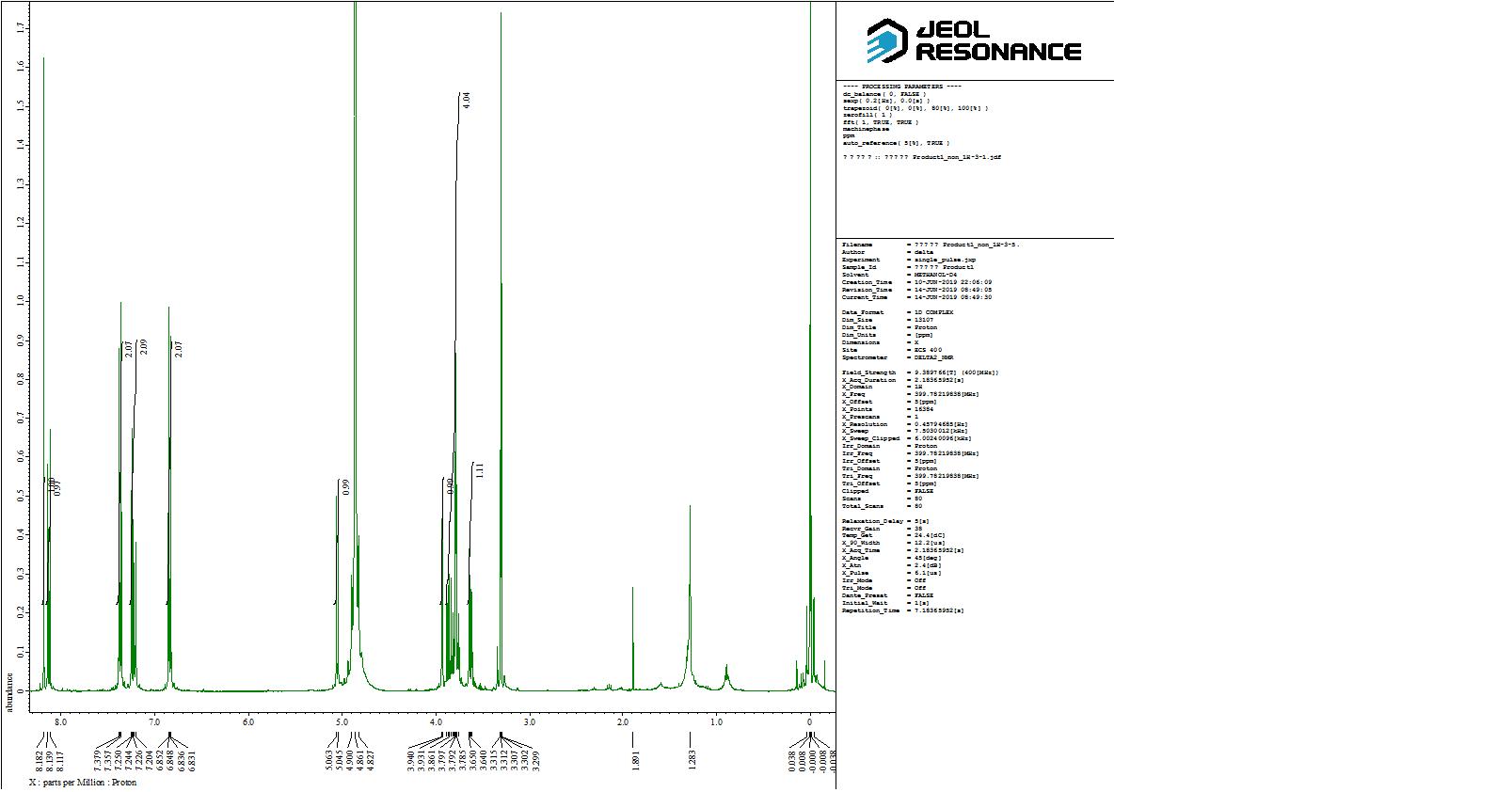

Supplement: Supplementary file 1 [file molecules-24-02975-s001.zip › supl/daidzein 7-galactoside 1H NMR.jpg]

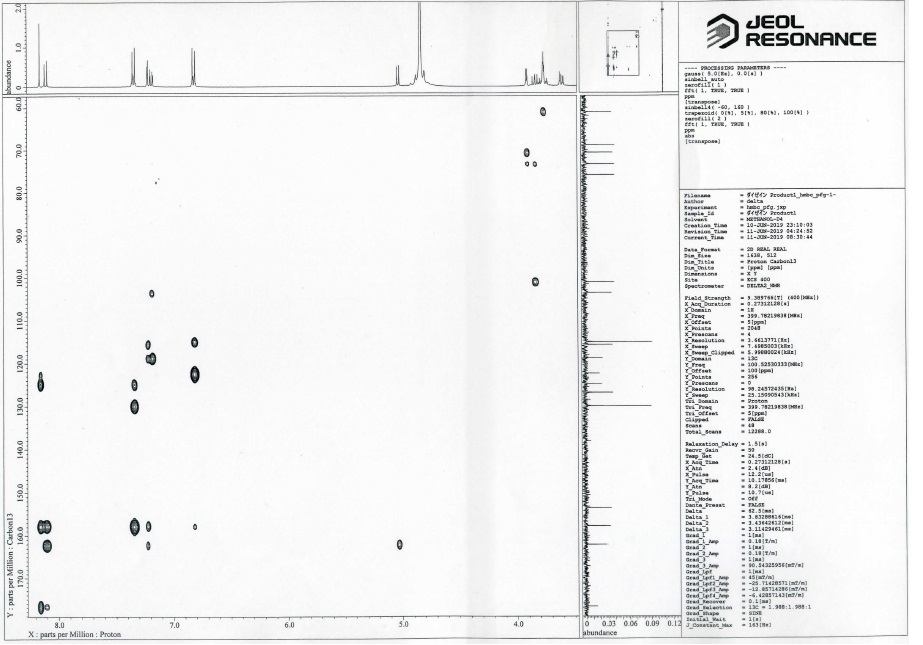

Supplement: Supplementary file 1 [file molecules-24-02975-s001.zip › supl/daidzein 7-galactoside hmbc.jpg]
